# Comparative phylogenetic analyses uncover the ancient roots of Indo-European folktales

**DOI:** 10.1098/rsos.150645

**Published:** 2016-01-20

**Authors:** Sara Graça da Silva, Jamshid J. Tehrani

**Affiliations:** 1 Faculty of Social Sciences and Humanities, Institute for the Study of Literature and Tradition, New University of Lisbon, Avenida de Berna, 26-C, Lisboa 1069-061, Portugal; 2 Department of Anthropology and Centre for the Coevolution of Biology and Culture, Durham University, Durham DH1 1LE, UK

**Keywords:** cultural evolution, Indo-European, folktales, oral tradition, phylogenetics

## Abstract

Ancient population expansions and dispersals often leave enduring signatures in the cultural traditions of their descendants, as well as in their genes and languages. The international folktale record has long been regarded as a rich context in which to explore these legacies. To date, investigations in this area have been complicated by a lack of historical data and the impact of more recent waves of diffusion. In this study, we introduce new methods for tackling these problems by applying comparative phylogenetic methods and autologistic modelling to analyse the relationships between folktales, population histories and geographical distances in Indo-European-speaking societies. We find strong correlations between the distributions of a number of folktales and phylogenetic, but not spatial, associations among populations that are consistent with vertical processes of cultural inheritance. Moreover, we show that these oral traditions probably originated long before the emergence of the literary record, and find evidence that one tale (‘The Smith and the Devil’) can be traced back to the Bronze Age. On a broader level, the kinds of stories told in ancestral societies can provide important insights into their culture, furnishing new perspectives on linguistic, genetic and archaeological reconstructions of human prehistory.

## 1. Introduction

Recent investigations into the evolution of cultural diversity suggest that relationships among many languages [1–4], social behaviours [5–7] and material culture traditions [8–10] often reflect deep patterns of common ancestry that can be traced back hundreds or even thousands of years. In this study, we explore these relationships in a universally important and richly documented cultural domain: storytelling [11,12]. Theories concerning possible relationships between storytelling traditions and the descent histories of populations have a long pedigree, and were central to the concerns of pioneering folklorists in the nineteenth century. For example, Wilhelm Grimm argued that the traditional German tales that he and his brother Jacob had compiled were remnants of an ancient Indo-European cultural tradition that stretched from Scandinavia to South Asia: ‘The outermost lines [of common heritage in stories] … are coterminous with those of the great race which is commonly called Indo-Germanic, and the relationship draws itself in constantly narrowing circles round the settlements of the Germans … It is my belief that the German stories do not belong to the northern and southern parts of our fatherland alone but that they are the absolutely common property of the nearly related Dutch, English and Scandinavians’ [13], p. 576.

To date, however, efforts to investigate the descent histories of narrative traditions have been complicated by two main problems. Firstly, tales are not only transmitted ‘vertically’ from ancestral populations to their descendants but also spread ‘horizontally’ between contemporaneous societies as a result of trade, conquest and the dissemination of literary texts, profoundly disrupting the neat concentric patterns of common heritage envisaged by Grimm [14,15]. Secondly, given that folktales have been mainly transmitted through oral means, there is scant evidence to investigate their origins and historical distributions using conventional literary-historical methods. While Grimm believed that many folktales were likely to be thousands of years old, only a tiny minority can be traced back to before the emergence of the literary fairy tale in the sixteenth and seventeenth centuries. This has led to intense debates about the presumed antiquity of traditional tales [16], with some researchers claiming that many canonical fairy tales may actually be relatively recent literary inventions [17,18].

Here, we tackle these problems using quantitative phylogenetic methods that were initially developed in biology and have been recently employed to investigate the relationships between population histories and a number of cultural phenomena, such as languages [1,2,4], marriage practices [7], political institutions [19] material culture [8–10,20] and music [21]. Phylogenetic methods have also been applied to folklore to analyse cross-cultural distributions of international tale types/variants, and examine their relationships to spatial, genetic and linguistic patterns [22–25]. This research suggests that similarities among folktale corpora are correlated with both population histories and geographical proximity. However, no study has yet attempted to disentangle the specific legacies of common descent and regional diffusion, or to investigate how far back lineages of vertical transmission can be traced. In this paper, we address these issues directly.

## 2. Material and methods

### 2.1 Data

Data for our study were sourced from the Aarne Thompson Uther (ATU) Index—a catalogue of over 2000 distinct, cross-culturally stable ‘international tale types’ distributed among more than 200 societies [26]. We focused on ‘Tales of Magic’ (ATU 300–ATU 749), a category of stories featuring beings and/or objects with supernatural powers. We concentrated on magic tales as they represent the largest and most widely shared group of tales, and because they include the canonical fairy tales, which have been the main focus of debates about the origins of folktales [16]. We recorded the presence/absence of each these tales (*n*=275) in 50 Indo-European-speaking populations represented in the ATU Index (electronic supplementary material, table S1). We selected these populations as both their oral traditions [15] and their phylogenetic relationships [2,3] have been more intensively studied than any other group of cultures.

### 2.2 Trees

Following previous phylogenetic comparative studies of cultural traits [7,19,27,28], we employed language trees as a model for population histories. This approach is based on the well-established correspondences between population dispersals and the diversification of linguistic lineages [27]. Language trees represent an especially suitable model for the study of folktale inheritance since the latter consists of verbally transmitted traditions.

Trees for our study were sourced from Bouckaert *et al.*’s [2,29] Bayesian phylogenetic analyses of Indo-European languages. First, we matched each population included in our dataset with one of Bouckaert *et al.*’s linguistic groups. Next, we pruned the trees to remove taxa for which there was no corresponding folktale corpus except Hittite, an ancient Anatolian population that spoke a language considered to be an outgroup of the Indo-European language family [2,3,7,30]. Hittite was retained to root the trees for the purposes of the analyses described below (electronic supplementary material, figure S1).

### 2.3 Testing for phylogenetic signal

To test for signatures of vertical transmission, we measured how well the distribution of each tale could be accounted for by the populations’ linguistic relationships using Fritz and Purvis’ *D* statistic [31]. *D* is a measure of phylogenetic signal that expresses the number of character changes in a binary trait on a tree scaled by two null distributions: one in which character states are randomly reshuffled among the tips of the tree, and one where the character evolves under a selectively neutral, Brownian model of evolution. A *D* of 0 indicates that the distribution of character states among the taxa is what would be expected for a neutral trait under a purely vertical mode of inheritance, while values approaching 1 approximate a phylogenetically random distribution. *D* scores lower than 0 imply greater levels of phylogenetic conservatism than would be anticipated under a Brownian model, while scores higher than 1 suggest overdispersion. The phylogenetic signal indicated by a *D* score can be statistically assessed by testing whether the number of character state changes required for a trait is significantly lower than would be expected by chance, based on the distribution of values returned by the random model.

*D* values for each tale in our sample were estimated using the phylo.d function in the caper package in *R* [32]. We simulated the evolution of each trait 1000 times under both null models on a majority-rules consensus tree, which was calculated from the tree sample and rooted using Hittite as an outgroup (electronic supplementary material, figure S1). Tales were coded as present or absent in each population based on the information contained in the ATU Index (electronic supplementary material, table S1). As no folktale data are available for Hittite and the program does not allow for missing data, all tales were initially coded as absent in the outgroup. The results of the analyses were then checked by re-analysing the data with states for Hittite coded as present.

### 2.4 Autologistic analyses

**Figure 1.**
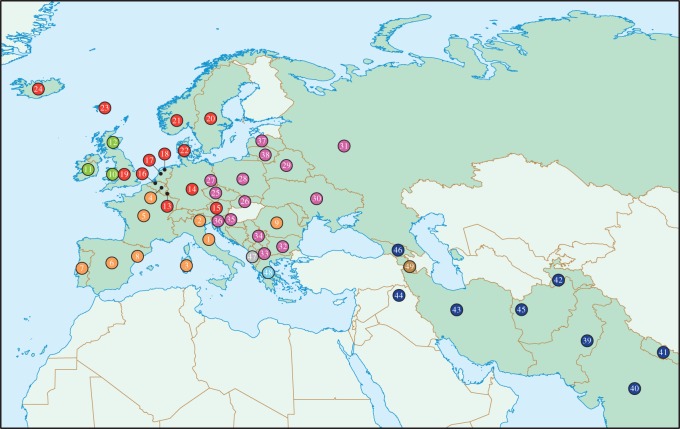
Approximate locations of Indo-European-speaking populations in Eurasia. Points are colour-coded by linguistic subfamily: red, Germanic; pink, Balto-Slavic; orange, Romance; green, Celtic; blue, Indo-Iranian; Turquoise, Hellenic; grey, Albanian; brown, Armenian. Numbers correspond to point references for populations listed in the electronic supplementary material, table S2.

Our second set of analyses tested whether phylogenetic signatures identified in the *D* analyses remained robust when accounting for the populations’ spatial relationships. Since many closely related Indo-European populations are also geographical nearest neighbours (figure 1), it is possible that the apparent non-random clumping of tales on the phylogeny may be the result of regional diffusion between societies. To address this issue, we employed an approach developed by Towner *et al.* [33] for fitting binary cultural traits to an autologistic model built on phylogenetic and spatial neighbour graphs (electronic supplementary material, figure S2). The model predicts the probability of a trait being present or absent in any given society from the state of the trait in its surrounding spatial and phylogenetic neighbours. The influences of these local dependencies are measured by parameters for phylogenetic (*λ*) and spatial (*θ*) proximity, with a level parameter (*β*) employed to control for frequency-of-occurrence. The likelihood of each parameter is estimated through MCMC simulations using the Gibbs sampler [34] to generate trait states (see [33] for a detailed explanation).

For the purposes of our analyses, we constructed a phylogenetic neighbour graph based on membership of the same linguistic subfamily (i.e. Romance, Germanic, Balto-Slavic, Celtic and Indo-Iranian) and a spatial neighbour graph based on distances between point references for each society (electronic supplementary material, table S2). The graphs included all the populations for which folktale data were available except for Romani, who, as a highly dispersed ethnic group, could not be identified with a specific geographical location. We sought to match the average number of spatial neighbours as closely as possible to the average number of phylogenetic neighbours, because a large disparity in the connectedness of the networks might confound any comparison of their effects on tale distributions [33]. We determined that linking societies located within a 1000 km radius of one another produced a spatial neighbour graph with a similar average number of neighbours (15.3) as the linguistic neighbour graph (13.6). There was substantial overlap between the two neighbour graphs, with 110 pairs of societies being both spatial and linguistic neighbours. Nevertheless, there was a sufficient number of unique spatial neighbour pairs (*n*=265) and unique linguistic neighbour pairs (*n*=224) to separate the effects of the two graphs on the tale distributions. Following initial tuning of parameter priors, 25 000 Gibbs realizations were sampled from 51 000 Markov chain Monte Carlo (MCMC) generations at an interval of two, with the first 1000 generations discarded as burn-in. The analyses were performed in *R* using the code written by Towner *et al.* [33].

### 2.5 Reconstructing ancestral states

**Figure 2.**
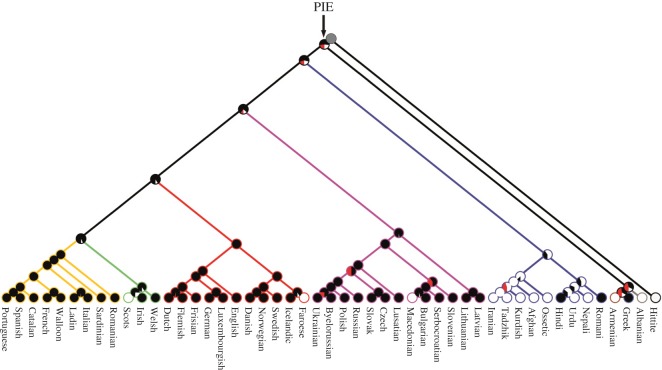
Reconstructing tale descent histories. Example of an ancestral state reconstruction, showing ATU 330 ‘The Smith and the Devil’ traced on a consensus tree derived from 1000 Bayesian language trees. The proportion of black shading in each internal node represents the average probability of the tale being present in the corresponding hypothetical ancestor across the tree sample. The proportion of red shading in each node represents the number of trees in which the corresponding hypothetical ancestor was absent. Branches are colour-coded by linguistic subfamily. The oldest ancestral node that was reconstructed, Proto-Indo-European, is labelled ‘PIE’.

To establish how far back shared folktales could be traced in Indo-European oral traditions, we mapped the evolutionary histories of the most phylogenetically conserved tales identified from the *D* and autologistic analyses using two models of discrete trait evolution implemented in Mesquite v. 3.02 [35]: (i) a Markov *k*-state one parameter model (Mk1), which estimates a single instantaneous rate of change for both gains and losses, given the distribution of the focal trait, a tree and set of branch lengths; (ii) an asymmetrical Markov *k*-state 2 parameter model (Mk2), which estimates separate rates for gains and losses on the tree. The most suitable model for each tale was selected on the basis of an asymmetrical likelihood ratio test. To incorporate uncertainty in Indo-European phylogenetic relationships and branch lengths, the tales were traced on every tree contained in our sample of 1000 Bayesian language phylogenies. Ancestral states were inferred for the nodes contained in a majority-rules consensus tree, which was rooted using Hittite as an outgroup. As no data on Hittite magic tales were available, trait states were coded as missing so that they did not bias the outcome of the analyses. The likelihood of any given tale having existed in a hypothetical ancestral population was calculated by estimating the average likelihood of the tale’s presence in the corresponding node across the tree, multiplied by the posterior probability of the node itself (i.e. its frequency in the tree sample; figure 2).

An additional set of Bayesian analyses were carried out on tales inferred as being potentially present in the populations’ hypothetical last common ancestor, ‘Proto-Indo-European’. We targeted this node for further investigation for two reasons: firstly, to test the support for the deepest reconstructions suggested by the analyses described above; and secondly, to control for the higher degree of phylogenetic uncertainty toward the root of the Indo-European language tree, which can be more effectively addressed within a Bayesian framework. Instead of calculating transition rates that maximize the likelihood of a trait distribution for each individual tree and then averaging the likelihood of it being present or absent at a particular node across the tree sample, the Bayesian approach estimates a posterior probability of ancestral states that integrates uncertainty about both transition rates and phylogenetic relationships simultaneously [28,36]. The posterior probability is obtained by recording ancestral states at regular intervals during a MCMC simulation, in which the trees and transition rates used to map the trait are sampled in proportion to their probabilities. We carried out the analyses using the Multistate model implemented in the software package BayesTraits v. 2.0 [36], using the same sample of 1000 Indo-European language trees and data on tale distributions from the ATU Index [26] as our previous analyses. Two sets of analyses were performed. The first estimated the posterior probability of each tale being present in Proto-Indo-European using the ‘most recent common ancestor’ command. The second analysis tested the relative support for each tale being present or absent by ‘fossilizing’ (i.e. fixing) the node in each state, and comparing the likelihood of the two models using Bayes Factors [37]. All the analyses employed uniform priors, the range of which was determined empirically following a maximum-likelihood analysis. The MCMC chains ran for 1 000 000 iterations, every 1000th of which was sampled into the posterior distribution following a burn-in period.

## 3. Results

*D* values for the 275 tales in our sample ranged from −2.06 to 3.9, with 100 tales exhibiting a higher degree of phylogenetic clumping than would be expected by chance (*α*=0.05) (electronic supplementary material, table S3). These results were stable whether trait states in the outgroup taxon, Hittite, were coded as present or absent.

**Figure 3.**
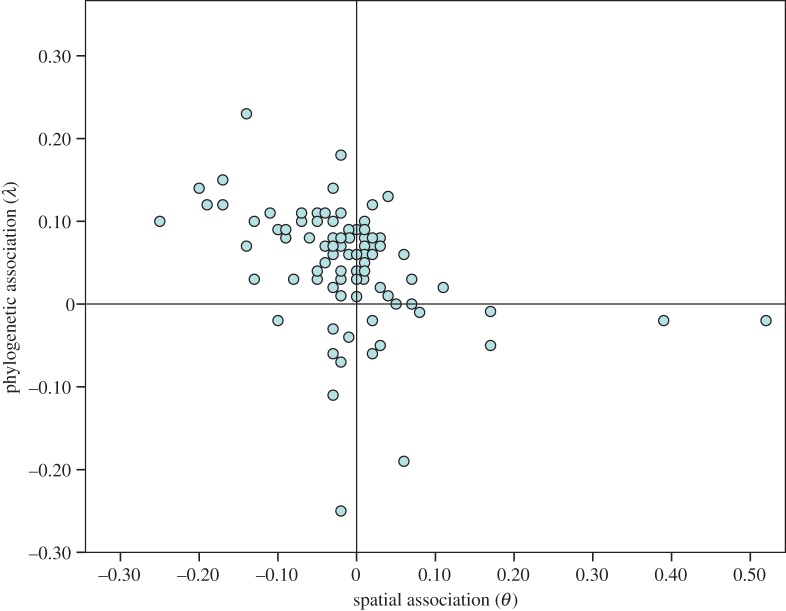
Estimates for phylogenetic and spatial association in the autologistic analyses. Scatter plot of phylogenetic (*λ*) and spatial (*θ*) parameters estimated for 100 tales that returned a strong phylogenetic signal in the *D* analyses when fitted to the autologistic model.

When fitted to the autologistic model, the distributions of 81 of the 100 tales that returned a significant phylogenetic signal in the *D* analysis were positively associated with the populations’ linguistic affiliations (electronic supplementary material, table S4). Only 36 tales were positively associated with spatial proximity, while in 56 cases tales were found to be *less* likely to be shared among societies who are spatial neighbours. Overall, the autologistic analyses suggested that vertical transmission was more important than horizontal transmission in 76 tales (figure 3 and table 1).

**Table 1.** Effects of phylogenetic and spatial association on tale distributions estimated by the autologistic model. Numbers in the cells represent the number of tales affected positively, negatively or neutrally by spatial (Spa) and phylogenetic associations (Phy) among populations.

	Spa +	Spa −	Spa 0	
Phy +	25	48	8	81
Phy −	9	8	0	17
Phy 0	2	0	0	2
	36	56	8	

**Figure 4.**
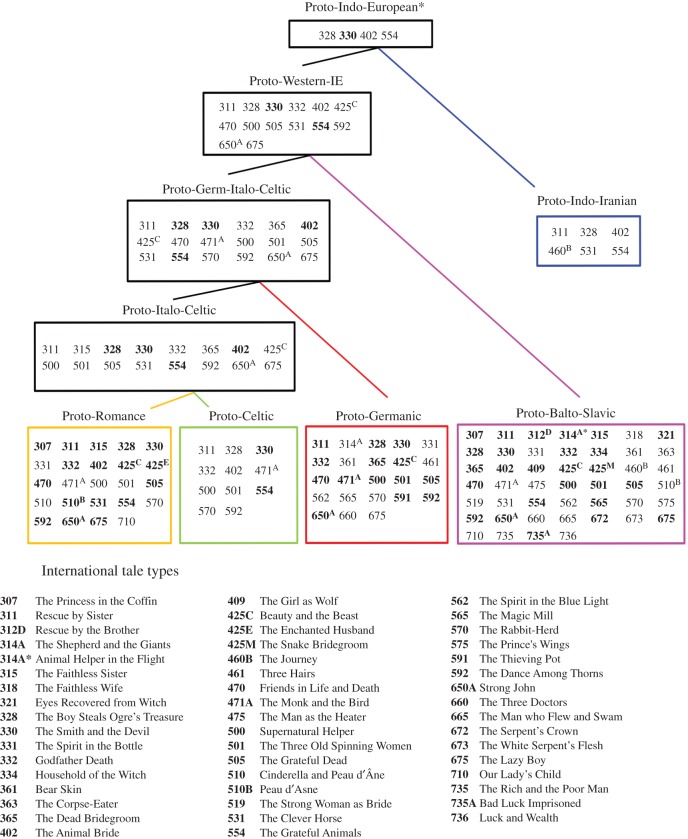
Estimated contents of ancestral tale corpora. Reconstruction of ancestral Indo-European tale corpora based on analyses of the 76 most phylogenetically conserved tales. Tales contained in each box were reconstructed with a more than 50% likelihood of being present in the corresponding ancestral tale corpus whereas tales in bold represent cases where tales could be securely reconstructed (greater than or equal to 70%). Full results for the ancestral state reconstructions are provided in the electronic supplementary material, table S5. Asterisks denote reconstructions in Proto-Indo-European are based on the results of Bayesian analyses (table 2).

Ancestral states were inferred for the 76 most phylogenetically conserved tales identified in the *D* and autologistic analyses. All the tales except two could be traced back to at least one of the hypothetical common ancestors represented in figure 2 with a probability of greater than 50%, 71 of which could be inferred with a high degree of confidence (greater than or equal to 70% likelihood) [28] (electronic supplementary material, table S5). Fifty tales were reconstructed as having been present in the last common ancestor of one or more major Indo-European sub-families with a likelihood more than 50%, with 31 at 70% or higher (figure 4). Nineteen tales could be traced back to even earlier ancestral populations with a likelihood of more than 50%, including four that were inferred in the last common ancestor of all the populations included in the sample (Proto-Indo-European). However, only a small proportion of tales could be securely reconstructed in these groups, with four tales in Proto-Italic-Celtic and Proto-Italic-Celtic-Germanic, two in Proto-Western-European and no tales in Proto-Indo-European surpassing 70% likelihood.

The Bayesian ancestral state reconstructions failed to support the presence of three out of the four tales that were tentatively inferred in Proto-Indo-European (table 2). However, the analyses reconstructed one tale, ATU 330 ‘The Smith and the Devil’, in this corpus, with a posterior probability of 87%. A fossil test returned positive support for the presence of ATU 330 (Bayes Factor 3.59).

**Table 2.** Results of the Bayesian analyses of Proto-Indo-European tales. Posterior probabilities for the presence/absence of tales reconstructed in Proto-Indo-European were obtained from a most recent common ancestor analysis, performed in BayesTraits (v. 2) [36]. The relative support for each possibility was further assessed by a fossil test. Bayes Factor support for the presence of each tale was evaluated using the interpretive framework suggested by Kass & Rafftery [37].

tale	*p* (present)	*p* (absent)	Bayes Factor support for presence (interpretation)
ATU 330	0.87	0.13	3.59 (‘positive’)
ATU 554	0.5	0.5	1.91 (‘weak’)
ATU 328	0.39	0.61	0.69 (‘weak’)
ATU 402	0.5	0.5	0.35 (‘weak’)

## 4. Discussion

Our analyses of the distributions of Tales of Magic among Indo-European-speaking populations bear out the observations of previous researchers concerning the complex spatial and historical patterning of the international folktale record [14,15,24,25]. Nevertheless, they show that it is still possible to uncover deep signatures of common descent in the folktale traditions of related populations. The results of the *D* analyses suggested that a substantial number of tales (100 of 275) exhibit significant correlations with linguistic relationships that are consistent with vertical processes of cultural inheritance. The majority of these correlations (76 out of 100) remained robust even after accounting for spatial relationships among linguistically related Indo-European groups in the autologistic analyses. In fact, in most of these cases, spatial proximity appears to have had a negative effect on the tales’ distributions, suggesting that societies were more likely to reject than adopt these stories from their neighbours.

The latter finding contrasts with previous research that reports much stronger evidence for the spatial diffusion of folktales between neighbouring populations. A study by Ross *et al.* [24] found that similarities among European variants of the tale ‘The Kind and Unkind Girls’ (ATU 480) are strongly correlated with geographical proximity independently of linguistic relationships, but not vice versa. Another more recent study by Ross & Atkinson [25] suggests that the distributions of shared tale types among Arctic hunter–gather societies are predicted by both geographical and linguistic associations, with the former being more influential. However, it is important to emphasize that we only compared spatial versus phylogenetic effects for tales that had already been screened for a phylogenetic signal (in order to determine whether that signal was genuine). It is highly plausible that horizontal transmission played a much greater role in the tales whose distributions were not predicted by linguistic relationships in the *D* analyses—which included ATU 480 ‘The Kind and Unkind Girls’, consistent with Ross *et al.*’s [24] findings. This raises a more general question about why populations seem to readily adopt some tales from their neighbours, while apparently rejecting others. Theoretical studies of cultural evolution suggest that patterns of cultural diversity are often shaped by parochial transmission biases (e.g. conformism, neophobia) that inhibit the exchange of information between groups and preserve local distinctions [6,38–40]. However, relatively little work has examined the extent to which these biases target particular kinds of traits, or the circumstances under which they might be relaxed [9,41,42]. While the answers to these questions lie beyond the scope of this study, our findings regarding the differentiated phylogenetic and spatial distributions of folktales provide a rich context for further investigation into these problems.

The durability of the phylogenetic signatures returned by the *D* analysis and autologistic tests, highlighted by the ancestral state reconstructions, revealed the existence of shared ancestral traditions in each of the major clades of the Indo-European family (figure 4). The results of these analyses have major implications for current debates concerning the origins of Tales of Magic [16,17]. Whereas most folklorists since Grimm believe that written versions of fairy tales were originally derived from oral tradition, some literary scholars [17,18] have claimed that there is very little evidence to support the precedence of oral traditions over literary ones and argued that it is unlikely that these stories could have been transmitted intact for so many generations without the support of written texts. Our findings contradict the latter view, and suggest that a substantial number of magic tales have existed in Indo-European oral traditions long before they were first written down (electronic supplementary material, table S5). For example, two of the best known fairy tales, ATU 425C ‘Beauty and the Beast’ and ATU 500 ‘The Name of the Supernatural Helper’ (‘Rumplestiltskin’) were first written down in the seventeenth and eighteenth centuries [43]. While some researchers claim that both storylines have antecedents in Greek and Roman mythology [44,45], our reconstructions suggest that they originated significantly earlier. Both tales can be securely traced back to the emergence of the major western Indo-European subfamilies as distinct lineages between 2500 and 6000 years ago [2,3], and may have even been present in the last common ancestor of Western Indo-European languages (figure 4).

In general, the number of tales that could be inferred in ancestral tale corpora decreases as they approach the root of the tree, with a concomitant decline in the reliability of these reconstructions. Although fourteen tales were inferred as present in Proto-Western-Indo-European (more than 50% likelihood), only two had a likelihood of more than 70%. Four tales were inferred as having a greater than 50% likelihood of being present in Proto-Indo-European, none of which had a likelihood of more than 70%. While the phylogenetic signal of a tale is bound to be eroded over time by transmission errors, competition with other tales, population turnover and diffusion between groups, the reconstruction of very ancient Indo-European tale traditions is further problematized by the uncertainty associated with deeper nodes in the tree. Thus, whereas the hypothetical ancestors for Proto-Romance, Proto-Germanic, Proto-Celtic and Proto-Indo-Iranian have a posterior probability of 100% in our tree sample, the corresponding value for Proto-Western-Indo-European is 90%, falling to 77% for Proto-Indo-European. However, despite these limitations, we were able to trace the inheritance of several tales deep into Indo-European prehistory, securely reconstructing them in the tale corpora of Proto-Italo-Celtic (ATU 328, ATU 330, ATU 402 and ATU 554), Proto-Italo-Celtic-Germanic (ATU 328, ATU 330, ATU 402 and ATU 554) and Proto-Western-European (ATU 330 and ATU 554). Even more remarkably, the Bayesian analyses were able to infer the presence of one tale, ATU 330 ‘The Smith and the Devil’, in the last common ancestor of the Indo-European family, Proto-Indo-European (table 2).

In sum, the results of the ancestral state reconstructions demonstrate that phylogenetic comparative methods can yield penetrating insights into the contents of ancient tale corpora which are difficult to access using conventional literary-historical approaches. Of course, this does not diminish the value of excavating the literary record for evidence about the origins and development of oral tales. Indeed, research carried out in this vein can supply extremely useful means of cross-checking the results of comparative phylogenetic analyses. For example, research into tale types and motifs in Graeco-Roman, Germanic and Celtic mythology support the antiquity of many of the magic tales that we reconstructed in ancestral Indo-European populations (electronic supplementary material, table S5). These data provide useful materials for further efforts to validate our findings. Ancient variants could be used to calibrate phylogenetic analyses of specific tale types [22,23] in the same way that ancient languages are used to date the origins of linguistic families [2,3]. Hypotheses concerning the descent history of a given international type (e.g. ATU 330) could then be tested against the structure of phylogenetic relationships and estimated root age inferred from different historical and cultural versions of the tale.

In some cases, it may also be possible to evaluate inferences about ancestral tale corpora in relation to other sources of information about past societies, such as historical, archaeological, linguistic and genetic data. Our findings regarding the origins of ATU 330 ‘The Smith and the Devil’ are a case in point. The basic plot of this tale—which is stable throughout the Indo-European speaking world, from India to Scandinavia—concerns a blacksmith who strikes a deal with a malevolent supernatural being (e.g. the Devil, Death, a jinn, etc.). The smith exchanges his soul for the power to weld any materials together, which he then uses to stick the villain to an immovable object (e.g. a tree) to renege on his side of the bargain [26]. The likely presence of this tale in the last common ancestor of Indo-European-speaking cultures resonates strongly with wider debates in Indo-European prehistory, since it implies the existence of metallurgy in Proto-Indo-European society. This inference is consistent with the so-called ‘Kurgan hypothesis’, which links the origins of the Indo-European language family to archaeological and genetic evidence of massive territorial expansions made by nomadic pastoralist tribes from the Pontic steppe 5000–6000 years ago [3,46–48]. The association of these peoples with a Bronze Age technological complex, as reconstructed from material culture data [49] and palaeo-linguistic inferences of PIE vocabulary (which include a putative word for metal, *a*⌢*ios*) [50], suggests a plausible context for the cultural evolution of a tale about a cunning smith who attains a superhuman level of mastery over his craft. By contrast, the presence of this story in PIE society appears to be incompatible with the alternative ‘Anatolian hypothesis’ of Indo-European origins. The latter proposes a much earlier and more gradual process of demic diffusion associated with the spread of agriculture from Neolithic Anatolia 8000–9000 years ago [51]—prior to the invention of metallurgy. However, it should be noted that according to some variants of the model [2,52], the lineage leading to all surviving Indo-European languages may have diverged from the now extinct Anatolian languages as recently as 7000–5500 B.C.E, a range which overlaps with the earliest archaeological evidence for smelting at numerous sites in Eurasia [53]. Consequently, a Bronze Age origin for ATU 330 seems plausible under both major models of Indo-European prehistory.

On a more general level, this example highlights how the kinds of stories told in ancient populations often reflect broader features of their cultures. While the content of ATU 330 is most obviously relevant to the technological capabilities of Proto-Indo-European society, anthropologists have long speculated that folktales may preserve other kinds of information about the ancestral contexts in which they originated, such as social organization, subsistence practices and religion [14,54]. Comparative phylogenetic methods provide a powerful set of tools with which to investigate these hypotheses more scientifically. We anticipate that future studies in this area will not only shed new light on the origins of fairy tales, myths, legends and other types of traditional narrative, but also offer novel and complementary perspectives on archaeological, genetic and linguistic reconstructions of the past.

## Supplementary Material

Table S1. Data Matrix: Cross-Cultural Distributions of Indo-European Magic Tales

## Supplementary Material

Supplementary Material: Contains Tables S2-S5 and Figures S1 & 2.

## Data accessibility

The datasets supporting this article have been uploaded as part of the electronic supplementary material.
