# Supplementary material for: Comparative phylogenetic analyses uncover the ancient roots of Indo-European folktales
Source: R Soc Open Sci. 2016 Jan 20;3(1):150645. doi: 10.1098/rsos.150645 (PMC4736946; doi:10.1098/rsos.150645)
Supplement: Supplementary Material: Contains Tables S2-S5 and Figures S1 & 2. [file rsos150645supp2.pdf]

Supplementary Materials

Table S1. Data Matrix: Cross-Cultural Distributions of Indo-European Magic Tales (included separately as an Excel file).

Table S2. Data used to build linguistic and spatial neighbour graphs……………………………… page 2

Table S3. Results of the Phylogenetic Signal Tests…………………………………………………… pages 3-13

Table S4. Autologistic Results………………………………………………………………………………… pages 14-18

Table S5. Ancestral States Reconstructions……………………………………………………………. pages 19-20

Figure S1. Figure S1. Indo-European language tree……………………………………………………..page 21

Figure S2. Linguistic and spatial neighbour graphs…………………………………………………….page 22

Supplementary Material References……………………………………………………………………………page 23

Table S2. Data used to build linguistic and spatial neighbour graphs

ID	Language	Subfamily	Longitude	Latitude
1	Italian	Romance	12,619597	43,070702
2	Ladin	Romance	11,874274	46,552591
3	Sardinian	Romance	8,935919	40,113209
4	Walloon	Romance	4,871985	50,467388
5	French	Romance	2,398782	47,081012
6	Spanish	Romance	-3,70379	40,416775
7	Portuguese	Romance	-8,632141	39,916775
8	Catalan	Romance	0,22649	40,30618
9	Romanian	Romance	24,152069	45,792784
10	Welsh	Celtic	-3,540195	52,447794
11	Irish	Celtic	-7,882514	53,22524
12	Scottish	Celtic	-3,729711	56,704361
13	Luxembourgish	Germanic	6,102779	49,752829
14	German	Germanic	11,02988	50,984768
15	Austrian	Germanic	14,24033	47,566855
16	Flemish	Germanic	3,717424	51,054342
17	Dutch	Germanic	5,117778	52,091667
18	Frisian	Germanic	5,799913	53,201233
19	English	Germanic	-1,890401	52,486243
20	Swedish	Germanic	15,22	59,319
21	Norwegian	Germanic	8,0049805	59,4901441
22	Danish	Germanic	9,536354	55,711311
23	Faroese	Germanic	-6,790982	62,007864
24	Icelandic	Germanic	-18,816667	64,816667
25	Czech	Slavic	14,68737	49,783782
26	Slovak	Slavic	19,643818	48,806073
27	Lusatian	Slavic	14,332868	51,756311
28	Polish	Slavic	19,455983	51,759249
29	Byelorussian	Slavic	27,566667	53,9
30	Ukrainian	Slavic	32,059767	49,444433
31	Russian	Slavic	37,6173	55,755826
32	Bulgarian	Slavic	25	42,25
33	Macedonian	Slavic	21,7411	41,5186
34	Serbian	Slavic	20,6875	43,723611
35	Croatian	Slavic	16,842309	45,898797
36	Slovenenian	Slavic	14,505751	46,056947
37	Latvian	Slavic	23,721354	56,651109
38	Lithuanian	Slavic	23,957728	55,288131
39	Pakistani	Indo-Iranian	71,468703	30,198381
40	Indian	Indo-Iranian	77,412615	23,259933
41	Nepali	Indo-Iranian	83,6	28,266667
42	Tadzhik	Indo-Iranian	69,525007	37,611453
43	Iranian	Indo-Iranian	52,369444	33,376111
44	Iraqui	Indo-Iranian	43,13	36,34
45	Afghan	Indo-Iranian	62,6031	34,49112
46	Ossetian	Indo-Iranian	43,972658	42,211671
47	Albanian	Albanian	19,818698	41,327546
48	Greek	Greek	22,4349	38,895973
49	Armenian	Armenian	45,18	39,76

The phylogenetic neighbour graph was based on membership of Indo-European subfamilies. The spatial neighbour graph linked populations located within a 1,000km radius of one another (based on centroid longtitude/latitude point references).

Table S3. Results of the Phylogenetic Signal Tests

Tale Type	Tale Name	D Statistic	*p*
300	The Dragon-Slayer	0.5700289	0.07
300A	The Fight on the Bridge	-0.4295884	0**
301	The Three Stolen Princesses	0.860333	0.33
301D	The Princess's Ring	0.2474298	0.04*
302	The Ogre's Heart in the Egg	0.7029113	0.13
302B	Life Dependent on a Sword	0.317801	0.04*
302C*	The Magic Horse	0.1712921	0.01**
303	The Twins or Blood Brothers	0.8043904	0.20
303A	Brothers Seek Sisters as wives	1.12156	0.66
304	The Dangerous Night-Watch	0.8780354	0.28
305	The Dragon's Heart-Blood as Remedy	1.329315	0.76
306	The Danced-out Shoes	1.198711	0.81
307	The Princess in the Coffin	0.28697	0**
310	The Maiden in the Tower	0.845941	0.24
311	Rescue by Sister	0.37459	0.01**
311B*	The Singing Bag	-1.005553	0**
312	Maiden-Killer (Bluebeard)	0.8188669	0.21
312A	The Rescued Girl	1.009628	0.48
312 C	The Rescued Bride	-2.05695	0**
312D	Rescue by the Brother	0.4153654	0.01**
313	The Magic Flight	0.5403695	0.06
313E*	The Sister's Flight	-0.4199701	0**

314	Goldener	0.7704425	0.11
314A	The Shepherd and the Three Giants	0.4358487	0.01**
314A*	Animal as Helper in the Flight	-0.7784967	0**
315	The Faithless Sister	0.5895249	0.03*
315 A	The Cannibal Sister	0.2684868	0.02*
316	The Nix of The Mill-Pond	0.9467939	0.37
317	The Tree that Grows up to the Sky	0.9660919	0.43
318	The Faithless Wife	0.5854899	0.05*
321	Eyes Recovered from Witch	-0.4230627	0**
322*	Magnetic Mountain Draws Everything to it	0.2200897	0.14
325	The Magician and his Pupil	1.038093	0.53
325*	The Sorcerer’s Apprentice	0.5080664	0.07
325**	Sorcerer Punished	0.2603893	0.24
326	The Youth Who Wanted to Learn What Fear is	0.6760554	0.11
326A*	Soul Released from Torment	0.6300508	0.06
326B*	The Youth and the Corpse	0.342718	0.04*
327	The Children and the Ogre	1.251181	0.85
327 A	Hansel and Gretel	0.7121716	0.12
327B	The Brothers and the Ogre	0.9616881	0.43
327C	The Devil (Witch) Carries the Hero Home in a Sack	0.9678057	0.41
327D	The Kiddelkaddelkar	1.242051	0.69
327F	The Witch and the Fisher Boy	-0.8212877	0**
327G	The Brothers at the Witch’s House	0.5745983	0.14
328	The Boy Steals the Ogre’s Treasure	0.2137311	0**
328A	Jack and the Beanstalk	0.3995604	0.20
328*	Three Giants with One Eye	0.5974897	0.20

328A*	Three Brothers Steal Back the Moon, Sun and Star	0.745798	0.31
329	Hiding from the Princess	0.8423354	0.23
330	The Smith and the Devil	0.06303866	0**
331	The Spirit in the Bottle	0.5893224	0.04*
332	Godfather Death	0.4787329	0.03*
332C*	Immortality Won through Betrayal of Death	1.894583	0.92
333	Little Red Riding Hood	1.256891	0.88
334	Household of the Witch	0.1719302	0**
335	Death's Messengers	0.949248	0.37
360	Bargain of the Three Brothers with the Devil	0.4988557	0.03*
361	Bear Skin	0.3846092	0.01**
361*	The Wolf with an Iron Head	0.2184486	0.15
362*	The Devil's Kindness	0.5066329	0.15
363	The Corpse-Eater	0.2253085	0.01**
365	The Dead Bridegroom carries off his Bride	0.5010155	0.03*
366	The Man from the Gallows	0.5031197	0.02*
368C*	The Death of the Cruel Stepmother	-0.1874915	0.12
369	The Youth on a Quest for his Lost Father	1.905131	0.92
400	The Man on a Quest for his Wife	0.9639815	0.40
401A*	The Soldiers in the Enchanted Castle	0.5149237	0.15
402	The Animal Bride	0.3069275	0.02*
402*	The Princess who Scorned an Unloved Suitor	1.22715	0.60
402A*	The Princess transformed into a Toad	1.297548	0.79
403	The Black and the White Bride	0.8167514	0.25
403C	The Substituted Bride	-0.2676684	0.02*
404	The Blinded Bride	0.8139693	0.23

405	Jorinde and Joringel	1.019561	0.50
406	The Cannibal	1.16867	0.63
407	The Girl as Flower	0.723221	0.12
408	The Three Oranges	0.9132528	0.33
409	The Girl as Wolf	-0.2491807	0**
409A	The Girl as Goat	1.009398	0.47
409A*	The Girl as Snake	-0.4316819	0**
409B*	The Promised Supernatural Wife	0.6923873	0.27
410	Sleeping Beauty	0.9513547	0.39
410*	The Petrified Kingdom	0.2520497	0.05*
411	The King and the Lamia	0.6783143	0.30
412	The Maiden (Youth) with a Separable Soul in a Necklace	-1.058891	0**
413	The Stolen Clothing	0.204817	0.03*
425	The Search for the Lost Husband	1.212231	0.82
425A	The Animal as Bridegroom	1.042346	0.55
425B	Son of the Witch	0.8924959	0.30
425C	Beauty and the Beast	0.4470139	0.02*
425D	The Vanished Husband	1.022611	0.50
425E	The Enchanted Husband sings Lullaby	0.08038305	0**
425M	The Snake as Bridegroom	-0.0001889632	0**
425*	The Insulted Bridegroom Disenchanted	1.219523	0.64
426	The Two Girls, the Bear, and the Dwarf	0.9074143	0.34
430	The Donkey	1.26022	0.85
431	The House in the Forest	0.9259195	0.35
432	The Prince as Bird	0.8089887	0.22
433B	King Lindorm	0.9376437	0.38

434	The Stolen Jewellery	0.3161727	0.18
434*	The Diver	3.665587	0.68
440	The Frog King or Iron Henry	0.2569959	0.01**
441	Hans My Hedgehog	0.773295	0.18
442	The Old Woman in the Forest	1.306124	0.68
444*	Enchanted Prince Disenchanted	1.441667	0.87
449	Sidi Numan	0.8078112	0.21
450	Little Brother and Little Sister	0.9261542	0.36
451	The Maiden who seeks her Brothers	0.6254541	0.07
452B*	The Sisters as Oxen	-0.9295624	0.02*
459	The Make-Believe Son (Daughter)	2.098858	0.85
460A	The Journey to God (Fortune)	0.6999888	0.10
460B	The Journey in Search of Fortune	0.336861	0.01**
461	Three Hairs from the Devil's Beard	0.2936121	0**
462	The Outcast Queens and the Ogress Queen	-0.03992126	0**
465	The Man Persecuted because of his Beautiful Wife	0.7977386	0.19
467	The Quest for the Wonderful Flower	0.647741	0.14
470	Friends in Life and Death	0.4419852	0.02*
470A	The Offended Skull	0.6485333	0.06
470B	The Land Where No One Dies	1.220442	0.81
471	The Bridge to the Otherworld	0.7097094	0.12
471A	The Monk and the Bird	0.2498829	0**
475	The Man as the Heater of Hell's Kettle	0.2192871	0**
476	Coal turns into Gold	1.331745	0.82
476*	In the Frog's House	0.2237879	0.04*
476**	Midwife in the Underworld	0.4387203	0.06

480	The Kind and the Unkind Girls	0.9470672	0.38
480A	Girl and the Devil in a Strange House	-0.082178	0**
480A*	Three Sisters set out to Save their Little Brother	-1.154356	0**
480C*	Transporting White Bread to Hell	1.010727	0.51
480D*	Tales of the Kind and Unkind Girls	1.177571	0.67
485	Borma Jarizhka	-0.4293035	0.02*
485B*	The Power of Drunkness	1.2035	0.61
500	The Name of the Supernatural Helper	0.1499802	0**
500*	The Monster Reveals the Riddle	1.043576	0.52
501	The Three Old Spinning Women	0.2531381	0**
502	The Wild Man	0.8828908	0.30
503	The Gift of the Little People	0.8729432	0.26
505	The Grateful Dead	0.3190476	0.01**
506*	Prophecy Escaped	-0.1508933	0.02*
507	The Monster's Bride	0.7178382	0.15
510	Cinderella and Peau d'Âne	0.3293585	0.02*
510A	Cinderella	0.8814071	0.29
510B	Peau d'Asne	0.5502516	0.03*
510B*	The Princess in the Chest	1.466346	0.79
511	One-Eye, Two-Eyes, Three-Eyes	0.8997079	0.32
513	The Extraordinary Companions	1.35282	0.92
513A	Six Go Through the Whole World	0.8743066	0.30
513B	The Land and Water Ship	1.066017	0.60
514	The Shift of Sex	0.873917	0.27
514**	A Young Woman Disguised as a Man is Wooed by the Queen	-0.5522485	0**
515	The Shepherd Boy	1.126143	0.53

516	Faithful John	0.6562563	0.07
516C	Amicus and Amelius	0.01367831	0**
517	The Boy Who Understands the Language of Birds	0.4897682	0.03*
518	Men Fight over Magic Objects	0.6505825	0.07
519	The Strong Woman as Bride (Brunhilde)	0.2595497	0.01**
530	The Princess on the Glass Mountain	0.8279799	0.22
530A	The Pig with the Golden Bristles	-0.5000603	0**
531	The Clever Horse	0.316228	0.01**
532*	The Magic Ox	1.08034	0.52
533	The Speaking Horsehead	1.327082	0.94
535	The Boy Adopted by Tigers	3.89561	0.75
537	The Flight on the Grateful Eagle	0.5593794	0.06
540	The Dog and the Sailor	0.18266010	0.09
545	The Cat as Helper	1.421465	0.95
545A	The Cat Castle	0.3376016	0.18
545B	Puss in Boots	1.311346	0.92
545A*	The Magic Castle	1.310016	0.92
545D*	The Pea King	1.301185	0.93
546	The Clever Parrot	2.374528	0.89
550	Bird, Horse and Princess	1.00069	0.47
551	Water of Life	0.8950713	0.32
552	The Girl who Married Animals	0.8003115	0.18
554	The Grateful Animals	0.4935952	0.04*
555	The Fisherman and his Wife	0.7866198	0.18
556F*	The Shepherd in the Service of a Witch	1.20542	0.72
559	Dungbeetle	0.8975381	0.31

560	The Magic Ring	0.9183016	0.37
561	Aladdin	0.6485731	0.07
562	The Spirit in the Blue Light	0.5330139	0.04*
563	The Table, the Donkey and the Stick	0.5285987	0.07
564	The Magic Providing Purse	0.725981	0.12
565	The Magic Mill	0.3216803	0.01**
566	The Three Magic Objects and the Wonderful Fruits	0.6325197	0.06
567	The Magic Bird-Heart	0.770512	0.17
567A	The Magic Bird-Heart and the Separated Brothers	0.6509102	0.11
569	The Knapsack, the Hat and the Horn	0.7164523	0.10
570	The Rabbit-Herd	0.5104798	0.02*
570A	The Princess and the Magic Shell	1.264863	0.68
570*	The Rat-Catcher	0.5223693	0.12
571	All Stick Together	0.836036	0.24
571B	Lover Exposed	0.8959416	0.30
571C	The Biting Doll	-0.1128121	0.01**
572*	The Barking Dog´s Head, the Striking Axe, etc.	-1.455774	0**
575	The Prince´s Wings	0.5122827	0.02*
576	The Magic Knife	1.298849	0.63
577	The King´s Tasks	0.7833048	0.20
580	Beloved of Women	0.5108169	0.03*
585	Spindle, Shuttle and Needle	-0.3701517	0.03*
590	The Faithless Mother	0.930038	0.37
591	The Thieving Pot	0.4685782	0.04*
592	The Dance Among Thorns	0.3613994	0.01**
593	Fiddevav	0.5175396	0.03*

594*	The Magic Bridle	1.089264	0.53
610	The Healing Fruits	0.9851429	0.45
611	The Gifts of the Demons	0.5307095	0.09
612	The Three Snake-Leaves	0.6548059	0.08
613	The Two Travellers	1.006413	0.47
650A	Strong John	0.3795928	0.01**
650B	The Quest for a Strong Companion	0.7778074	0.25
650C	The Youth who Bathed Himself in the Blood of a Dragon	-0.215422	0.01**
652	The Prince Whose Wishes Always Come True	0.7337531	0.14
653	The Four Skilful Brothers	0.9392327	0.39
653A	The Rarest Thing in the World	1.12186	0.69
653B	The Suitors Restore the Maiden to Life	1.366981	0.76
654	The Three Agile Brothers	0.4059535	0.02*
655	The Wise Brothers	0.3342787	0.01**
660	The Three Doctors	0.2702455	0**
664*	The Soldier Hypnotises the Innkeeper	-0.09727507	0.02*
665	The Man who Flew like a Bird and Swan like a Fish	0.4760786	0.02*
666*	Hero and Leander	1.367742	0.79
667	The Wood Spirit´s Foster-Son	0.3585237	0.05*
670	The Man who Understands Animal Languages	0.9989682	0.46
670A	The Woman Who Understands Animal Languages	0.9205802	0.41
671	The Three Languages	1.001862	0.50
671D*	To Die Next Day	0.7419502	0.24
671E*	A Magic Boy	1.013439	0.49
672	The Serpent´s Crown	-0.1446616	0**
672D	The Stone of the Snake	-0.192147	0**

672B*	Expelling Snakes	0.5209615	0.09
672C*	Testimony of the Serpent	1.694736	0.72
673	The White Serpent´s Flesh	0.1436674	0**
674	Incest Averted by Talking Animals	1.302571	0.68
675	The Lazy Boy	0.2526243	0**
677	Iron is More Precious than Gold	-0.3955285	0**
677*	Below the Sea	-1.29468	0.16
678	The King Transfers His Soul to a Parrot	-0.07911055	0.08
681	Relativity of Time	1.654546	0.83
682	Meditation on the Trinity	2.14578	0.99
700	Thumbling	0.8259203	0.26
701	The Giant´s Toy	0.1199105	0**
703*	The Artificial Child	-0.1405671	0**
704	Princess on the Pea	0.461862	0.07
705A	Born From Fruit (Fish)	0.7744834	0.16
705A*	The Banished Wife	-0.4811908	0.03*
706	The Maiden Without Hands	1.025762	0.50
706B	The Chaste Nun	1.219153	0.68
706C	The Father who Wanted to Marry his Daughter	0.8236181	0.25
706D	St. Wilgefortis and Her Beard	1.857168	0.91
707	The Three Golden Children	0.9355507	0.39
708	The Wonder Child	0.6355706	0.09
709	Snow White	0.8061164	0.19
709A	The Sister of Nine Brothers	2.584905	0.98
710	Our Lady´s Child	0.576397	0.04*
711	The Beautiful and the Ugly Twin Sisters	0.4399553	0.04*

712	Crescentia	1.038394	0.53
713	The Mother Who Did Not Bear Me but Nourished Me	0.1543963	0.09
715	Demi-Cock	0.7801143	0.17
715A	The Wonderful Rooster	-0.2953609	0.01**
716*	The Unbearable Satiety	-0.6926108	0.01**
720	The Juniper Tree	0.8514215	0.26
725	Prophecy of Future Sovereignty	0.784082	0.17
726	The Three Old Men	1.295418	0.90
729	The Merman´s Golden Axe	1.163729	0.74
735	The Rich Man’s and the Poor Man´s Fortune	0.1455137	0**
735A	Back Luck Imprisoned	-0.4454177	0**
736	Luck and Wealth	0.3352391	0.01**
736A	The Ring of Polycrates	1.445495	0.99
737	Who will be her Future Husband?	-0.06100311	0.01**
737B*	The Lucky Wife	-0.3602013	0**
739*	The Luck-Bringing Animal	-0.02972965	0.01**
740**	The Brother (the Poor Man) About to Hang Himself Finds a Treasure	0.4075615	0.06
745	Hatch-Penny	0.5987659	0.07
745A	The Predestined Treasure	0.6480321	0.07

Phylogenetic signal of each Magic Tale type, measured using Fritz and Purvis' D statistic [1]. Tales that returned a significantly stronger phylogenetic signal than chance are highlighted in grey, with corresponding p values provided in the last column (* p <0.05, ** p= <0.01).

Table S4. Autologistic Results

Tale type	Tale name	Phylogenetic Association λ	Spatial Association θ
**300A**	The Fight on the Bridge	0.05	-0.04
**301D**	The Princess's Ring	0	0.05
**302B**	Life Dependent on a Sword	0.01	0.04
**302C***	The Magic Horse	-0.06	-0.03
**307**	The Princess in the Coffin	0.07	0.02
**311**	Rescue by Sister	0.09	0
**311B***	The Singing Bag	-0.05	0.17
**312C**	The Rescued Bride	-0.02	0.52
**312D**	Rescue by the Brother	0.1	-0.13
**313E***	The Sister's Flight	0.03	0.009
**314A**	The Shepherd and the Three Giants	0.04	0
**314A***	Animal as Helper in the Flight	0.02	-0.03
**315**	The Faithless Sister	0.1	-0.03
**315A**	The Cannibal Sister	0.03	0.07
**318**	The Faithless Wife	0.08	-0.06
**321**	Eyes Recovered from Witch	0.14	-0.03
**326B***	The Youth and the Corpse	-0.03	-0.03
**327F**	The Witch and the Fisher Boy	0.03	-0.05
**328**	The Boy Steals the Ogre's Treasure	0.08	-0.02
**330**	The Smith and the Devil	0.12	0.02
**331**	The Spirit in the Bottle	0.07	-0.04
**332**	Godfather Death	0.11	-0.02
**334**	Household of the Witch	0.11	-0.05

**360**	Bargain of the Three Brothers with the Devil	-0.04	-0.01
**361**	Bear Skin	0.08	-0.03
**363**	The Corpse-Eater	0.09	-0.1
**365**	The Dead Bridegroom carries off his Bride	0.08	0.03
**366**	The Man from the Gallows	0.06	-0.01
**402**	The Animal Bride	0.09	0
**403C**	The Substituted Bride	-0.25	-0.02
**409**	The Girl as Wolf	0.01	-0.02
**409A***	The Girl as Snake	0.03	0
**410***	The Petrified Kingdom	-0.05	0.03
**412**	The Maiden (Youth) with a Separable Soul in a Necklace	0.18	-0.02
**413**	The Stolen Clothing	0.03	-0.02
**425C**	Beauty and the Beast	0.08	0.01
**425E**	The Enchanted Husband sings Lullaby	0.07	0.03
**425M**	The Snake as Bridegroom	0.06	-0.03
**440**	The Frog King or Iron Henry	0	0.07
**452B***	The Sisters as Oxen	-0.02	0.39
**460B**	The Journey in Search of Fortune	0.07	0.01
**461**	Three Hairs from the Devil’s Beard	0.08	-0.02
**462**	The Outcast Queens and the Ogress Queen	0.02	0.11
**470**	Friends in Life and Death	0.1	0.01
**471A**	The Monk and the Bird	0.04	0.01
**475**	The Man as the Heater of Hell’s Kettle	0.12	-0.17
**476***	In the Frog’s House	0.04	-0.05
**480A**	Girl and the Devil in a Strange House	0.04	-0.02

**480A***	Three Sisters set out to Save their Little Brother	-0.04	-0.01
**485**	Borma Jarizhka	-0.19	0.06
**500**	The Name of the Supernatural Helper	0.09	0.01
**501**	The Three Old Spinning Women	0.06	0.01
**505**	The Grateful Dead	0.11	-0.04
**506***	Prophecy Escaped	-0.009	0.17
**510**	Cinderella and Peau d'Âne	0.07	0.03
**510B**	Peau d'Âne	0.05	0.01
**514****	A Young Woman Disguised as a Man is Wooed by the Queen	0.13	0.04
**516C**	Amicus and Amelius	0.08	-0.009
**517**	The Boy Who Understands the Language of Birds	-0.02	0.02
**519**	The Strong Woman as Bride (Brunhilde)	0.05	-0.04
**530A**	The Pig with the Golden Bristles	0.07	-0.02
**531**	The Clever Horse	0.09	-0.01
**554**	The Grateful Animals	0.04	0.01
**562**	The Spirit in the Blue Light	0.07	-0.03
**565**	The Magic Mill	0.08	-0.009
**570**	The Rabbit-Herd	0.1	-0.07
**571C**	The Biting Doll	0.1	0.01
**572***	The Barking Dog´s Head, the Striking Axe, etc.	0.08	-0.09
**575**	The Prince´s Wings	0.08	-0.02
**580**	Beloved of Women	0.09	-0.1
**585**	Spindle, Shuttle and Needle	0.23	-0.14
**591**	The Thieving Pot	0.11	-0.11
**592**	The Dance among Thorns	0.08	0.02

**593**	Fiddevav	-0.07	-0.02
**650A**	Strong John	0.06	0.02
**650C**	The Youth who Bathed Himself in the Blood of a Dragon	-0.02	-0.1
**654**	The Three Agile Brothers	0.03	-0.13
**655**	The Wise Brothers	-0.01	0.08
**660**	The Three Doctors	0.09	-0.09
**664***	The Soldier Hypnotises the Innkeeper	0.009	0
**665**	The Man Who Flew Like a Bird and Swan Like a Fish	0.1	-0.05
**667**	The Wood Spirit´s Foster-Son	0.03	-0.08
**672**	The Serpent´s Crown	0.14	-0.2
**672D**	The Stone of the Snake	0.07	-0.14
**673**	The White Serpent´s Flesh	0.1	-0.25
**675**	The Lazy Boy	0.1	-0.03
**677**	Iron is More Precious than Gold	-0.06	0.02
**701**	The Giant´s Toy	0.12	-0.19
**703***	The Artificial Child	0.1	-0.05
**705A***	The Banished Wife	0.06	0.06
**710**	Our Lady´s Child	0.06	0
**711**	The Beautiful and the Ugly Twin Sisters	0.06	-0.01
**715A**	The Wonderful Rooster	0.02	0.03
**716***	The Unbearable Satiety	-0.11	-0.03
**735**	The Rich Man’s and the Poor Man´s Fortune	0.09	0.01
**735A**	Back Luck Imprisoned	0.11	-0.07
**736**	Luck and Wealth	0.06	0
**737**	Who Will Be Her Future Husband?	0.15	-0.17

**737B***	The Lucky Wife	0.04	0.01
**739***	The Luck-Bringing Animal	0.03	0

Effects of phylogenetic and spatial association in predicting cross-cultural distributions of 100 tales that returned a strong phylogenetic signal in the D analysis, estimated in the autologistic model [2].

Table S5. Ancestral States Reconstructions

Tale Type	Tale Name	Model	PIE	PII	PWIE	PBS	PGIC	PG	PIC	PC	PR	More Recent Common Ancestor*	Earliest Literary Record
300A	The Fight on the Bridge	MK1										Latvian-Lithuanian (0.93)	The larger tale cycle ATU 300 'The Dragon Slayer' is attested to in Greek myth, including Perseus' fight with the sea monster Ketos [3].<br>Widely recorded since the 6th century in hagiographies of St. George [4].
307	The Princess in the Coffin	MK1				**0.82**					**0.96**		Anderson links the type to a miracle tale told by Phlegon of Tralles in the second century [5].
311	Rescue by the Sister	MK1		0.54	0.51	**0.71**	0.58	**0.73**	0.58	0.56	**0.85**	Irish-Scottish (0.82)	Cognate tales recorded in a 5th century hagiography and possibly earlier tales of Minos of Crete [5]. The classic literary version of the general type is Perrault's "Bluebeard" (ATU 312A) from 1697.
312D	Rescue by the Brother	MK1				**0.7**						Norwegian-Danish (0.86);<br>Irish-Scottish (0.91)	Like ATU 311, this tale is considered a part of the Bluebeard family of tales (see above) [5].
313E*	The Sister's Flight	MK1										Latvian-Lithuanian (0.94);<br>Russian-Ukrainian (0.93)	A variant of international type 313 'The girl as helper in the her's flight', which occurs in European literary collections since the Rennaissance, including Basile's *Pentamerone* [6].
314A	The Shepherd and the Three Giants	MK1						0.54				Czech-Slovakian (0.74);<br>Frisian-Dutch (0.75)	A variant of international type 314 'The youth transformed into a horse'. A version of the story is recorded in a 12th century French manuscript Robert le Diable [7]. Elements of the tale occur in Straparola's story "Guerrino" from 'Le piacevoli notti' from the sixteenth century, where they are combined with type 501 'The wild man' [6, 8].
314A*	Animal as Helper in the Flight	MK2				**0.85**							See above (314A).
315	The Faithless Sister	MK1				**0.78**			0.51		**0.88**	Luxembourgish-German (0.93)	na
318	The Faithless Wife	MK1				0.52							Ancient Egyptian narrative "Anubis and Batu" [9]. The manuscript has been in existance for over 3000 years [10].
321	Eyes Recovered from Witch	MK1				**0.85**							A member of the Baba Yaga family of tales, recorded in Slavic countries since the mid 18th century [11].
327F	The Witch and the Fisher Boy	MK2										Latvian-Lithuanian (0.90);	A member of type 327, which includes the famous tale "Hansel and Gretel". Versions of 327 have existed in print since the late Middle Ages [8].
328	The Boy Steals the Ogre's Treasure	MK1	0.51	0.66	0.64	**0.80**	**0.75**	**0.87**	**0.76**	0.69	**0.99**		Possible cognate in the Sumerian tale of Bilgames and the Netherworld [5].Depicted in a 14th century misericord from Oxford, England [4].
330	The Smith and the Devil	MK1	0.54		**0.77**	**0.97**	**0.91**	**1**	**0.92**	**0.9**	**1**		Ancient Greek myth of Sysyphos and Thantos, the god of death [3]. Christianized versions of the tale have been known in Europe since the 1500s and were the subject of popular chapbooks from the early 1700s [6].
331	The Spirit in the Bottle	MK1				0.56		0.54			0.54	Swedish-Danish (0.81);<br>Irish-Scottish (0.73)	Recorded since the Middle Ages, most famously in The Arabian Nights [6]
332	Godfather Death	MK1			0.59	**0.86**	0.68	**0.8**	0.69	0.63	**0.97**		Recorded since around 1300 [6].
334	Household of the Witch	MK1				**0.7**							na
361	Bear Skin	MK1				0.55		0.55				Irish-Scottish (0.73);<br>Lusatian-Ukrainian (0.83)	Literary versions since the 17th century [6].
363	The Corpse-Eater	MK1				0.64						Latvian-Lithuanian (0.90);<br>Swedish-Danish (0.93)	na
365	The Dead Bridegroom carries off his Bride	MK1				**0.8**	0.56	**0.93**	0.52				18th century ballad [4].
402	The Animal Bride	MK1	0.52	0.53	0.63	**0.87**	**0.70**		**0.70**	0.64	**0.97**	Irish-Scottish (0.94);<br>Frisian-Flemish (0.76)	Literary version 'The White Cat' by Madame d'Aulnoy, 1710 [6].
409	The Girl as Wolf	MK2				**0.75**							na
409A*	The Girl as Snake	MK1										Russian-Ukrainian (0.93);<br>Latvian-Lithuanian (0.94)	na
412	The Maiden (Youth) with a Separable Soul in a Necklace	MK1										Urdu-Hindi (0.99)	na
413	The Stolen Clothing	MK2										Urdu-Hindi (0.70)	na
425C	Beauty and the Beast	MK1			0.52	**0.85**	0.6	**0.94**	0.57		**0.97**		The earliest version of the general tale type 425, 'Search for the Lost Husband' is found in Lucius Apuleius's Golden Ass, 2nd century century and in the Sanskrit *Panchatantra* from the 3rd century. The modern literary tradition begins with Straparola's tale "Galeotto" from *Le Piacevoli Notti* (1550) and Gabrielle-Suzanne de Villeneuve's classic novella "La Belle et la Bete" from 1740 [8].
425E	The Enchanted Husband sings Lullaby	MK1									**0.95**	Serbocroatian-Macedonian (0.88)	The earliest version of the tale cycle 425, 'Search for the Lost Husband' is found in Lucius Apuleius's Golden Ass, 3rd century C.E. [5].
425M	The Snake as Bridegroom	MK2				**0.80**							The earliest version of the tale cycle 425, 'Search for the Lost Husband' is found in Lucius Apuleius's Golden Ass, 3rd century C.E. [5].
460B	The Journey in Search of Fortune	MK1		0.51		0.61						Russian-Ukrainian (0.88);<br>Serbocroatian-Macedonian (0.88);<br>Urdu-Hindi (0.87);<br>Latvian-Lithuanian (0.79)	na
461	Three Hairs from the Devil's Beard	MK1				0.6		0.67				Catalan- Portuguese-Spanish (0.84)<br>Lusatian-Ukrainian (0.96);<br>Faroese-Danish (0.80);<br>Frisian Flemish (0.76);<br>Catalan- Portuguese-Spanish	Cuneiform fragments of the Assyrian myth of Izdubar represent a possible and extremely early version of the tale type from c. 2000 BCE [5].
470	Friends in Life and Death	MK1			0.51	**0.85**	0.62	**0.96**	0.61		**0.96**		Attested in the legend of Don Juan from 1630s onward [6].
471A	The Monk and the Bird	MK1				0.53	0.52	**0.75**		0.53	0.51	Latvian-Lithuanian (0.75);<br>Norwegian-Danish (0.91);<br>German-Dutch (0.81);<br>Catalan-Spanish-Portuguese (0.85)	Part of a wider tale cycle (ATU 471 'Brideg to Another World') known in the Seven Sages tradition with early Indian parallels from 5th century [6].
475	The Man as the Heater of Hell's Kettle	MK1				0.66						Walloon French (0.97);<br>Latvian-Lithuanian (0.81);	na
476*	In the Frog's House	MK1										Czech-Slovakian (0.95)	na
480A	Girl and the Devil in a Strange House	MK1										Russian Ukrainian (0.93);<br>Latvian Lithuanian (0.94)	Cognate tales belonging to the same general type (ATU 480) occur in Basile's *Pentamerone* and Perrault's *Histoires ou contes du temps passé* from the seventeenth century [6].
500	The Name of the Supernatural Helper	MK1			0.52	**0.8**	0.62	**0.98**	0.60	0.6	0.61	Catalan-Spanish (0.86);<br>Irish-Scottish (0.94);	Cognate tale summarised in Dio of Halicarnassus' 'Roman Antiquities', 1st century [5]. The modern literary tradition is traced to Basile's tale "The Seven Bits of Bacon Rind" from Lo cunto de li cunti (1634) and Marie-Jeanne L'Heritier's "Ricdin-Ricdon" from 1705 [8].
501	The Three Old Spinning Women	MK1				**0.7**	0.57	**0.87**	0.56	0.56	0.67	Catalan-Spanish-Portuguese (0.84);<br>Irish-Scottish (0.88);<br>Italian-Ladin (0.88)	Literary versions in Germany from 1669 [6].
505	The Grateful Dead	MK1			0.58	**0.86**	0.64	**0.88**	0.59		**0.7**		Apocryphal book of Tobit (c.200 - 170 BC) and medieval romances of Rittertreue and Sir Amadas [4].
510	Cinderella and Peau d'Âne	MK2									0.54	Flemish-Frisian (0.72)	The 1st century Graeco-Egyptian tale of Rhodopis the Courtesan is frequently cited as the earliest recognisable variant of the Cinderella cycle, dating from 1st century BCE [3,5].
510B	Peau d'Asne	MK1				0.57					**0.81**	Lusatian-Ukrainian (0.89);<br>Latvian-Lithuanian (0.72)	A version of the tale ("Dolarice") occurs in Straparola's 'Le piacevoli notti' from the sixteenth century . The French writer Nöel du Fail mentions the existence of a potential Donkeyskin tale ("Cuir d'Asnette") in Breton oral tradition in 1547, although he does not provide details of the plot [7].
514**	A Young Woman Disguised as a Man is Wooed by the (	MK1										Catalan-Spanish-Portuguese (0.93)	na
516C	Amicus and Amelius	MK1										Czech Slovak (0.93);<br>Italian-Portuguese (0.94)	na
519	The Strong Woman as Bride (Brunhilde)	MK1				0.56						Latvian-Lithuanian (0.89);<br>Russian-Ukrainian (0.92)	Versions of the tale are known in the Nordic myths of Brunhilde, and have been recorded in epic verse since medieval times, including the German epic *Nibelungenlied* [6]
530A	The Pig with the Golden Bristles	MK1										Latvian-Lithuanian (0.94);	
531	The Clever Horse	MK1		0.65	0.60	**0.86**	0.65		0.63		**0.95**	Faroese-Danish (0.81);<br>Urdu-Hindi (0.95)	Thompson traces some elements of the Clever Horse in some versions of the Tristam story, and one of its motifs back to the Ancient Egyptial tale 'The Two Brothers' [6]. Beecher also finds a connection with the Egyptian papyrus (1250 BC) containing the story "The Two Brothers" which was translated in 1852 [12].
554	The Grateful Animals	MK2	0.61	**0.79**	**0.71**	**0.85**	**0.78**		**0.78**	**0.77**	**0.91**	Swedish-Danish (0.87)	The third tale in *Gesta Romanorum, "The ingrato et gydone"*, focuses on the theme of the grateful animals (c. 1342) [13]
562	The Spirit in the Blue Light	MK1				0.57		0.53				Bulgarian-Macedonian (0.87);<br>Swedish-Danish (0.85);<br>Frisian-Flemish (0.70);	Part of a complex of tale types associated with Aladdin and the Lamp, incorporated into the One Thousand and One Nights by the French writer Antoine Galland in 1709 [6].
565	The Magic Mill	MK1				**0.75**		**0.80**				Irish-Scottish (0.89);<br>Spanish-Portuguese-Catalan (0.79)	Connected to the *Prose Edda* of Snorri Sturluson (around 1220) [9].
570	The Rabbit-Herd	MK1				0.61	0.51	0.56		0.52	0.51	Latvian-Lithuanian (0.71);<br>Bulgarian-Ukrainian (0.84);<br>Frisian-Flemish (0.70);<br>Irish-Scottish (0.77);<br>Catalan-Spanish-Portuguese (0.79)	Incorporated into the Fenian Cycle of British Isles legends in the Middle Ages [4].
571C	The Biting Doll	MK2										Catalan-Spanish-Portuguese (1)	na
572*	The Barking Dog´s Head, the Striking Axe, etc.	MK1										Latvian-Lithuanian (0.98)	na
575	The Prince´s Wings	MK1				0.64						Urdu-Hindi (0.86);<br>Latvian-Lithuanian (0.78);<br>Serbocroatian-Ukrainian (0.85);<br>Frisian-Flemish (0.70)	
585	Spindle, Shuttle and Needle	MK1										Irish-Scottish (1)	na
591	The Thieving Pot	MK2						**0.77**					Cognate of the Egyptian myth of Isis and Osiris [9]
592	The Dance Among Thorns	MK1			0.55	**0.83**	0.65	**0.93**	0.65	0.65	**0.83**		Told in Europe since the fifteenth century [9]

650A	Strong John	MK1			0.54	**0.83**	0.63	**0.9**	0.62		**0.92**		Connected to classical myths of the tasks of Herakles [6] and the legend of Achilles' heel [3].
654	The Three Agile Brothers	MK2										Frisian-Dutch (0.87);	A summary version of this tale is contained in the collection *Scala Celi* (c. 1300) by the French Dominican Johannes Gobii Junior [13].
660	The Three Doctors	MK1				0.62		0.52				Latvian-Lithuanian (0.81): Frisian-Dutch (0.95)	Can be traced back to one of the most famous medieval collections - *Gesta Romanorum* - whose earliest dated manuscript is from 1342 [13].
664*	The Soldier Hypnotises the Innkeeper	MK2										Russian - Ukrainian (0.86)	na
665	The Man who Flew like a Bird and Swan like a Fish	MK2				0.65						Slovenian- Ukrainian (0.78); Latvian-Lithuanian (0.75)	na
672	The Serpent´s Crown	MK1				**0.9**							na
672D	The Stone of the Snake	MK2										Slovenian-Ukrainian (0.76)	na
673	The White Serpent´s Flesh	MK2				0.59						Irish-Scottish (0.92)	Incorporated into the Fenian Cycle of British Isles legends in the Middle Ages [4].
675	The Lazy Boy	MK1			0.53	**0.79**	0.59	0.6	0.58		**0.95**	Faroese-Danish (0.83)	The tale appears in Straparola's 'Le piacevoli notti' from the sixteenth century (Thompson, 1977; Bottigheimer 2014). Danae's myth, described in Ovid's Book IV of the *Metamorphoses,* published around 8 C.E. [14].
701	The Giant´s Toy	MK2										English-Dutch (0.85)	na
703*	The Artificial Child	MK1										Latvian-Lithuanian (0.92);	na
710	Our Lady´s Child	MK1				0.51					0.54		Possibly related to the Greek myth of Gyges and Kroisos [3].
711	The Beautiful and the Ugly Twin Sisters	MK2										Icelandic-Norwegian (0.78); Irish-Scottish (0.84);	na
735	The Rich Man´s and the Poor Man´s Fortune	MK1				0.65						Latvian-Lithuanian (0.95); Serbocroatioan-Macedonian (0.94);	Occurs in an episode of the Greek myth of Jason and Medea - the death of Pelias [5].
735A	Back Luck Imprisoned	MK1				**0.88**							na
736	Luck and Wealth	MK1				0.64						Urdu-Hindi (0.93); Serbocroatian-Ukrainian (0.90)	A subtype of this tale occurs in a 16th century text on the Celtic legend of Maelgwyn [4].
737	Who will be her Future Husband?	MK1										Irish-Scottish (0.98)	na
737B*	The Lucky Wife	MK1										Latvian-Lithuanian (0.96); Serbocroatian-Macedonian (0.94)	na
739*	The Luck-Bringing Animal	MK1										Latvian Lithuanian (0.95)	na

Reconstruction of ancestral states for the 76 most phylogenetically conserved tales in eight ancestral Indo-European populations: Proto-Indo-European (PIE), Proto-Western-Indo-European (PWIE); Proto-Indo-Iranian (PII);Proto-Balto-Slavic (PBS);Proto-Germanic-Italo-Celtic (PGIC);Proto-Germanic (PG); Proto-Italo-Celtic (PIC); Proto-Celtic (PC), and Proto-Romance (PR).Cells in grey represent cases were a probability of <50% was observed. Values in bold refer to cases were a probability of 70% or over was noted are reported. Only values > 70% are reported for More Recent Common Ancestors. The last column includes information on the earliest literary references to the tales in the literary record. Three tales (ATU 580, ATU 366 and ATU 667) could not be securely reconstructed as present in any ancestral population and are therefore excluded from the table.

Figure S1. Indo-European language tree

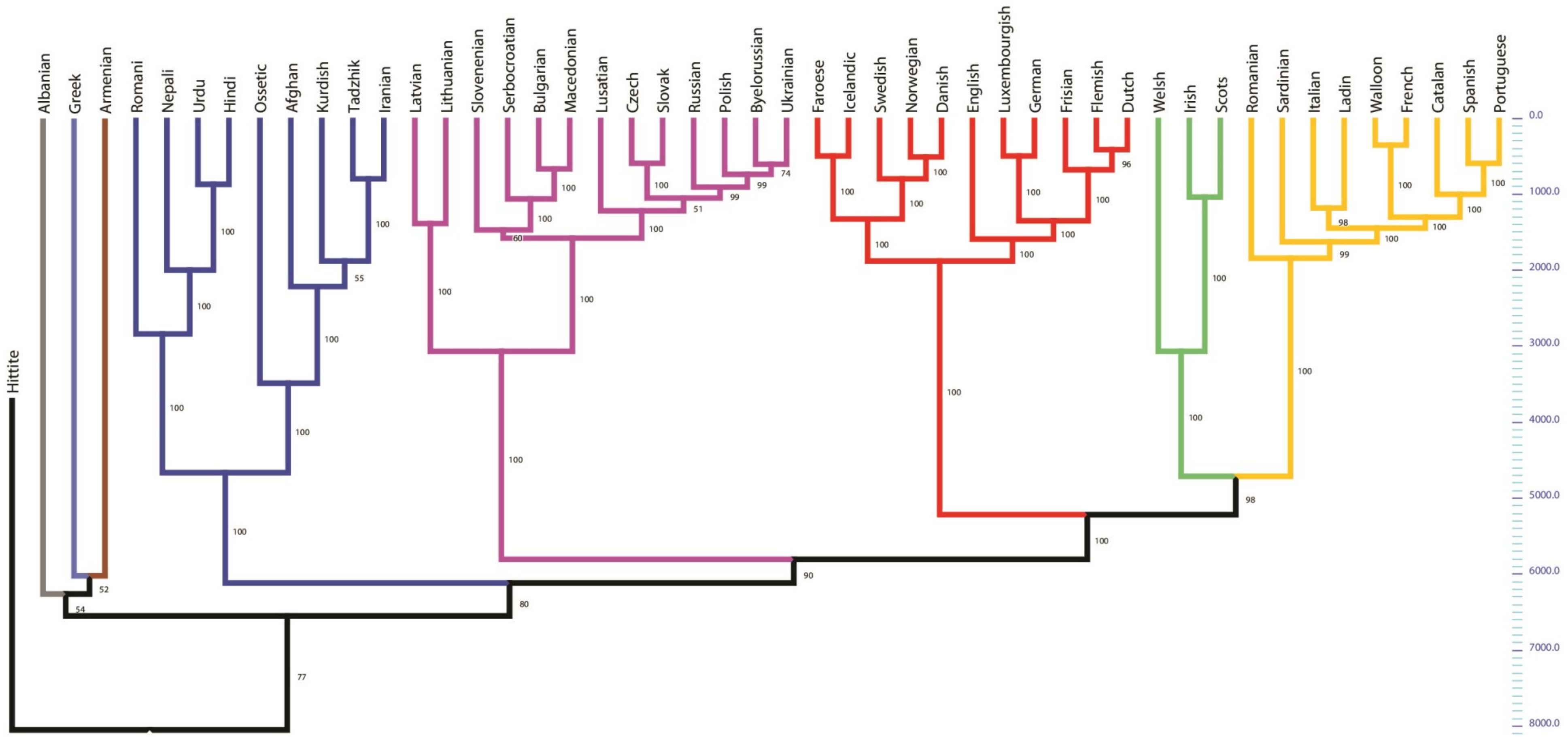

Time-calibrated majority-rules consensus tree calculated from the sample of 1,000 language trees. The trees were sourced from Bouckaert et al.'s [15, 16] Bayesian posterior distribution of Indo-European language trees and pruned to remove taxa not represented in the ATU Index except Hittite, which was retained to root the trees. The scale measures years from the present day. The numbers beneath internal nodes indicate posterior probabilities for the corresponding clade. Branches are colour-coded by linguistic subfamily: Red = Germanic; Pink = Balto-Slavic; Orange = Romance; Green = Celtic; Blue = Indo-Iranian; Turquoise = Hellenic; Grey = Albanian; Brown = Armenian.

Figure S2. Linguistic and spatial neighbour graphs

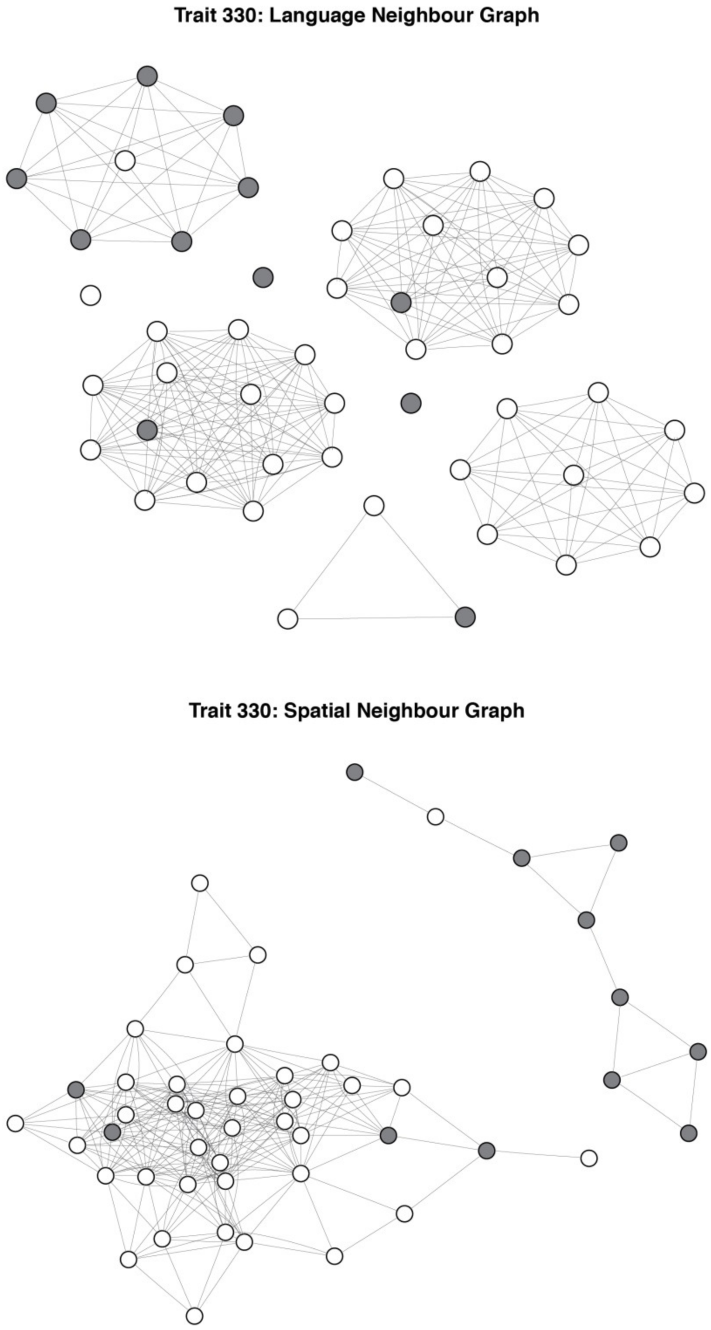

Example of a tale (ATU 330 'The Smith and the Devil') embedded in the spatial neighbour graph and the phylogenetic neighbour graph used in the autologistic analyses [2].

Supplementary Material References

1. Fritz S.A., Purvis A. 2010 Selectivity in Mammalian Extinction Risk and Threat Types: a New Measure of Phylogenetic Signal Strength in Binary Traits. Conservation Biology 24(4), 1042-1051.

2. Towner M., Grote M., Venti J., Borgerhoff Mulder M. 2012 Cultural Macroevolution on Neighbor Graphs. Human Nature 23(3), 283-305. (doi:10.1007/s12110-012-9142-z).

3. Hansen, W. 2002 *Ariadne's Thread: A Guide to International Tales Found in Classical Literature*. Ithaca & London, Cornell University Press.

4. Lindahl, C., McNamara, J. & Lindow, J. (eds.) 2000 *Medieval Folklore*. Oxford, Oxford University Press.

5. Anderson, G. 2000 *Fairytale in the Ancient World*. London and New York, Routledge.

6. Thompson, S. 1977 *The Folktale*. Berkeley, University of California Press.

7. Vaz da Silva, F. 2010 The Invention of Fairy Tales. *Journal of American Folklore*, 123: 398–425.

8. Zipes, J. 2013 *The Irresistible Fairy Tale: The Cultural and Social History of a Genre*. NJ, Princeton University Press.

9. Haase, D. (ed) 2008 *The Greenwood Encyclopedia of Folktales and Fairy Tales*. 3 vols. Westport, CT, Greenwood Publishing.

10. Maspero, G., El-Shamy, Hasan M. (eds.) 2004 *Popular Stories of Ancient Egypt*. Oxford, Oxford University Press.

11. Johns, A. 2004 *Baba Yaga: The Ambiguous Mother and Witch of the Russian Folktale*. Bern, Peter Lang.

12. Beecher, D. 2012 *The Pleasant Nights*, vol. 1. Toronto, University of Toronto Press.

13. Blamires, D. 1992 *Folktales and Fairy Tales in the Middle Ages*. Bulletin of the John Rylands University Library of Manchester 74 (1): 97–107.

14. Bottigheimer R. 2014 *Magic Tales and Fairy Tale Magic: From Ancient Egypt to the Italian Renaissance*. Palgrave, Macmillan.

15. Bouckaert R., Lemey P., Dunn M., Greenhill S.J., Alekseyenko A.V., Drummond A.J., Gray R.D., Suchard M.A., Atkinson Q.D. 2012 Mapping the Origins and Expansion of the Indo-European Language Family. Science 337(6097), 957-960. (doi:10.1126/science.1219669).

16. Bouckaert R., Lemey P., Dunn M., Greenhill S.J., Alekseyenko A.V., Drummond A.J., Gray R.D., Suchard M.A., Atkinson Q.D. 2013 Corrections and Clarifications. Science 342(6165), 1446. (doi:DOI: 10.1126/science.342.6165.1446-a)
